# Handgrip Strength and Current Smoking Are Associated with Cardiometabolic Risk in Korean Adolescents: A Population-Based Study

**DOI:** 10.3390/ijerph17145021

**Published:** 2020-07-13

**Authors:** Sae Yun Kim, Jung Soo Lee, Yeo Hyung Kim

**Affiliations:** 1 Department of Pediatrics, College of Medicine, The Catholic University of Korea, Seoul 06591, Korea; sysmile@gmail.com; 2 Department of Rehabilitation Medicine, College of Medicine, The Catholic University of Korea, Seoul 06591, Korea; drlee71@naver.com

**Keywords:** muscle strength, cardiovascular diseases, metabolic syndrome, adolescent, smoking

## Abstract

This study aimed to identify the independent association of handgrip strength and current smoking with cardiometabolic risk in adolescents. Data of 1806 adolescents (12–18 years) from the Korea National Health and Nutrition Examination Surveys were analyzed by complex samples logistic regression analyses. Handgrip strength was normalized by body weight into relative handgrip strength. A cardiometabolic risk index score was calculated from the z-scores of the following components: waist circumference, triglycerides, high-density lipoprotein cholesterol, glucose, and blood pressure. Relative handgrip strength showed an inverse association with high cardiometabolic risk, with an adjusted odds ratio of 8.5 (95% confidence interval [CI], 3.7–19.3) for boys and 5.7 (95% CI, 2.9–11.2) for girls on comparing the lowest and the highest age-and sex-specific quartiles of relative handgrip strength. The adjusted odds ratios for high cardiometabolic risk on comparing the second quartile and the highest quartile of relative handgrip strength were 3.9 (95% CI, 1.7–8.9) in boys and 2.6 (95% CI, 1.3–5.3) in girls. Current smoking was independently associated with high cardiometabolic risk in boys aged 15–18 years. These findings suggest the need to increase muscle strength in adolescents and reduce smoking in older boys to promote cardiometabolic health.

## 1. Introduction

Metabolic syndrome, an important healthcare burden worldwide, is a cluster of cardiometabolic risk factors associated with an increased risk of cardiovascular disease and type 2 diabetes [1]. Even though the clinical manifestations of metabolic syndrome usually appear in adulthood, evidence suggests that the origin of cardiovascular disease develops with the clustering of related risk factors since childhood [2]. Accordingly, early detection and interventions for adolescents with high cardiometabolic risk are essential to developing adequate public healthcare programs.

In pediatric populations, however, there is no consensus on the definitions of metabolic syndrome [3]. In addition, because children and adolescents tend to have no established cardiometabolic diseases, the prevalence of metabolic syndrome diagnosed from dichotomous risk factors is generally very low [4]. Therefore, metabolic syndrome cannot adequately represent the pediatric population at risk of cardiometabolic diseases. Considering these limitations, a combined cardiometabolic risk score reflecting the clustering of cardiometabolic risk factors has recently been suggested as a better measure of cardiovascular and metabolic health in adolescents than a single clinical feature [5,6,7,8,9].

Recently, muscular fitness has been regarded as an essential protective factor for health across the whole ages. The relationship between muscular strength and cardiovascular health is obvious in the elderly [10]. The association between muscular fitness and health benefit is also demonstrated in the pediatric population [11]. Weak muscular strength in the adolescent period has shown to be related to adverse cardiovascular health and even mortality in adulthood [12,13,14,15]. The gradual development of muscular strength occurs from 3 years of age [16]. Boys exhibit dramatic acceleration of muscular strength development until the end of puberty, and girls show a pronounced plateauing in late adolescence [17]. This natural physiologic development of muscular strength according to age and sex make the relationship between muscular strength and cardiometabolic health in adolescence more intricate than elderly people.

Handgrip (HG) strength is strongly associated with total muscle strength, and it is very conveniently measured [18]. Several epidemiological and clinical studies have found that weak HG strength is related to unfavorable cardiometabolic health in childhood and adolescence. A study in Colombia reported that poorer relative HG strength was associated with a worse metabolic risk profile, such as blood pressure, homeostatic model assessment (HOMA) index, triglyceride (TG), and a composite metabolic risk score in low–middle socioeconomic status schoolchildren [19]. The association between HG strength and metabolic syndrome have been suggested in European and Korean adolescents [8,20]. Some investigators in Colombia and Europe have been studied the cut-points of HG strength for detecting the presence of metabolic syndrome and elevated cardiometabolic risk index (CMRI) by using receiver operating characteristic (ROC) analyses [5,8]. Accordingly, these previous studies did not adjust for important confounders, such as height, smoking habits, and alcohol consumption, due to the focus of the study and statistical approach.

Considering the on-going physical growth and development of adolescents, numerous confounders can affect health outcomes in this population [21,22,23]. However, to date, the evidence on the association between HG strength and cardiometabolic risk in adolescents has been inferred by studies without adjustment for the confounding factors [5,20] or with minimal adjustment for cardiorespiratory fitness [8]. Therefore, the current study aimed to evaluate the association between cardiometabolic risk and age, sex, and body weight-adjusted HG strength in adolescents, while considering other potential confounders. In addition, although smoking has been suggested as an important risk factor for metabolic syndrome in adolescents as well as in adults [23], knowledge on the association between smoking habit and cardiometabolic risk is limited. Most previous studies on cardiometabolic risk in adolescents have not considered smoking behavior in the study design [5,8,19,20]. Therefore, the secondary objective was to determine the association of smoking behavior with cardiometabolic risk in adolescents.

## 2. Materials and Methods

### 2.1. Study Design and Participants

The present study was conducted using data from the Korea National Health and Nutrition Examination Surveys (KNHANES) VI and VII (2014–2017). The Korean Centers for Disease Control and Prevention (KCDC) collects health-related data by interviews and physical examinations every year. The KNHANES implements multi-stage, stratified, and clustered probability sampling to gather a representative sample of the community-dwelling population aged ≥1 year. All individuals signed a consent form. The Institutional Review Board at the KCDC approved the protocol. A detailed data profile and sampling method of KNHANES have been published elsewhere [24]. The approval of our university was not required for using a database with public access. The KCDC provides up-to-date information about the KNHANES on its website (https://knhanes.cdc.go.kr).

A total of 31,207 individuals (14,197 men and 17,010 women) participated during the study period of 2014–2017, and the response rate was 77.2%. Of these participants, 2298 adolescents aged 12–18 years were recruited (Figure S1). Finally, we included 1806 adolescents (952 boys and 854 girls) in the cross-sectional analyses after excluding the participants with missing data (*n* = 491) and an adolescent who was previously diagnosed with diabetes (*n* = 1).

### 2.2. Handgrip Strength Measurement

Maximal HG strength (kg) of both hands was measured three times alternately by trained nurses using a Takei digital dynamometer T.K.K.5401 (Takei, Niigata, Japan). The HG strength was assessed in participants excluding individuals with (1) amputations or paralysis of the arm, hand, or thumb; (2) casts or bandages in the wrist or hand; (3) wrist or hand surgery within 3 months; and (4) wrist or hand pain within one week. The standard position was standing upright with the arm straight down and the wrist and hand in a neutral position [25]. The HG strength of an individual was defined as the mean maximal HG strength from the 3 measurements of both hands. The relative HG strength was calculated as HG strength normalized by the body weight of each participant. The relative HG strength was categorized as age- and sex-specific quartiles.

### 2.3. Clinical and Biochemical Assessment

Sedentary time was assessed by asking adolescents to answer the question selected from the Global Physical Activity Questionnaire [26]: “How much time do you usually spend sitting or reclining on a typical day?” The participants were asked to include time spent at work, at home, getting to and from places, or with friends, but to exclude time spent sleeping. The sedentary time was categorized by age- and sex-specific quartiles. Smoking behavior and alcohol consumption were recorded in adolescents aged 12 years and above. The adolescents who reported smoking for more than 1 day during the past 30 days were regarded as current smokers [27]. Alcohol drinker was defined as someone who had two or more alcoholic drinks in a month [28].

Body weight, height, and waist circumference (WC) were measured to the nearest 0.1 kg or 0.1 cm. The body mass index (BMI) was obtained as follows: weight/height^2^ (kg/m^2^). Weight status was categorized according to Centers for Disease Control and Prevention in the United States standard categories, and the corresponding percentiles, as underweight, healthy weight, overweight, or obese (BMI <5th percentile, 5th–84th percentile, 85th–94th percentile, and ≥95th percentile, respectively) [29]. Age- and sex-specific weight status percentiles were calculated using the Korean National Growth Charts for adolescents [30].

Qualified nurses measured systolic and diastolic blood pressure (SBP and DBP, respectively) three times at 30-s intervals using a mercury sphygmomanometer (Baumanometer, Baum, Copiague, NY, USA) and a cuff sized according to the arm circumference. The final blood pressure value was recorded as the mean of the second and third measurements. After the participants had fasted for ≥8 h, the venous blood was sampled, processed immediately, and transported to a central laboratory. A Hitachi analyzer 7600-210 (Hitachi, Tokyo, Japan) was used for measuring fasting glucose, total cholesterol, high-density lipoprotein cholesterol (HDL-C), and TG levels.

### 2.4. Cardiometabolic Risk and Metabolic Syndrome Evaluation

The cardiometabolic risk of the adolescents was estimated using the CMRI score [5,6,7,8]. An age- and sex-standardized CMRI score was calculated for each participant, as previously defined: z-WC + z-TG + [(−1) × z-HDL-C] + z-glucose + z-SBP + z-DBP [5,6]. High cardiometabolic risk was defined as ≥1 standard deviation of the CMRI score. Metabolic syndrome was defined according to the criteria suggested by the International Diabetes Foundation (IDF). The IDF criteria, according to the age groups (12–15 years and 16–18 years), were applied [31].

### 2.5. Statistical Analysis

All statistical analyses were performed by complex-sample procedures of SPSS version 24 (IBM/SPSS Inc., Armonk, NY, USA) reflecting the sampling weights created to account for the complex survey design, non-response of the survey, and post-stratification. Because the KNHANES collects data of sample survey with a complex sampling design, sampling weights were considered in all analyses to estimate the information for the entire Korean population. The prevalence of metabolic syndrome and the participants with high cardiometabolic risk were obtained by complex sample descriptive procedures. Sensitivity and specificity were calculated from the weighted numbers of participants. The mean HG strength, according to age and sex, were obtained by complex sample general linear models. To compare the mean HG strength according to age, post hoc analyses, using the age of 18 years as the reference, were performed using general linear models for both sexes. The characteristics of adolescents, according to cardiometabolic risk, were analyzed by complex sample Student’s t-tests or the Chi-square tests. The associations between relative HG strength, smoking status, and other factors with cardiometabolic risk were assessed by complex sample multivariable logistic regression analyses. Height, sedentary time, and alcohol consumption, as well as age, sex, and body weight, were considered potential confounders [21,22,23,32].

## 3. Results

### 3.1. Characteristics of Adolescents

The unweighted and weighted prevalence of metabolic syndrome according to the IDF criteria was 2.7% (48/1806) and 2.7% (standard error [SE], 0.4%), respectively. The unweighted and weighted prevalence for adolescents with high cardiometabolic risk was 14.2% (257/1806) and 13.6% (SE, 0.9%), respectively. The sensitivity and specificity of the high cardiometabolic risk defined as ≥1 standard deviation of the CMRI score for metabolic syndrome were 97.0% and 88.8%, respectively. The characteristics of adolescents, according to the cardiometabolic risk, are shown in Table 1. For both sexes, the high cardiometabolic risk group was more likely to be obese and to have poor clinical and biochemical profiles than the low cardiometabolic risk group. For both sexes, the HG strength was significantly higher in the high cardiometabolic risk group than in the low cardiometabolic risk group, whereas the relative HG strength was lower in the high cardiometabolic risk group than in the low cardiometabolic risk group. The proportions of current smokers and alcohol drinkers and mean sedentary time were not different between the high and low cardiometabolic risk groups.

**Figure 1:**
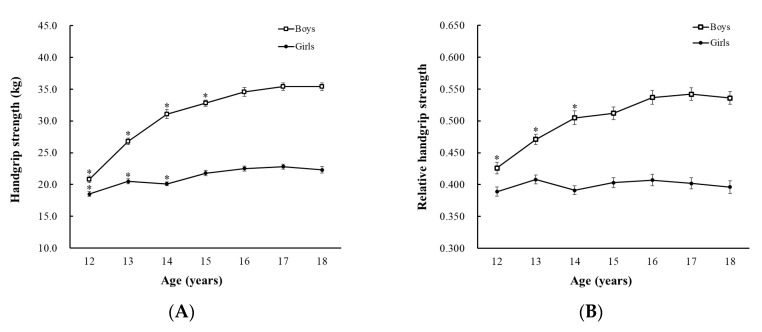
(**A**) Handgrip and (**B**) relative handgrip strength of the study population according to age and sex. Values are weighted means, and error bars represent the standard errors of the means. * *p* < 0.05 by post hoc analyses, age 18 years old as reference.

As seen in Figure 1, boys had higher HG strength and relative HG strength than girls (both *p* < 0.001). There was a significant increase in the HG strength according to age in both boys and girls. The mean increase was 14.6 kg for boys and 3.8 kg for girls for 6 years from 12 to 18 years. While the relative HG strength of boys increased with age, that of girls did not differ according to age. The HG strength of boys aged 12–15 was significantly lower than that of boys aged 18 years, and the relative HG strength of boys aged 12–14 was significantly lower than that of boys aged 18 years by post hoc analyses. The HG strength of girls aged 12–14 was significantly lower than that of girls aged 18 years. Meanwhile, the relative HG strength of girls in all ages was similar to that of girls aged 18. The data on HG strength and relative HG strength according to age and sex are presented in Table S1.

### 3.2. Association of Relative HG Strength and Current Smoking with Cardiometabolic Risk

An independent inverse association was found between age- and sex-specific relative HG strength and cardiometabolic risk (Table 2). For both sexes, the cardiometabolic risk was significantly higher in participants in the lowest quartile (Q1) and second-lowest quartile (Q2) than in those in the highest quartile (Q4) of age- and sex-specific relative HG strength. These negative associations remained significant even after adjusting for height, smoking status, alcohol drinking, and sedentary time. The cardiometabolic risk of participants in the third quartile (Q3) of relative HG strength was similar to that of participants in the highest quartile (Q4).

Because 94.9% (93/98) of the current smokers were aged 15 years or older, the study participants were stratified as young adolescents (12–14 years old) and older adolescents (15–18 years old). As shown in Table 3, negative associations between age- and sex-specific relative HG strength and cardiometabolic risk were present in both young and older adolescents. The adjusted odds ratios (ORs) of relative HG strength for high cardiometabolic risk were found to be higher in older adolescents than in young adolescents in both sexes. Current smoking showed an independent association with high cardiometabolic risk in older boys (adjusted OR 2.5; 95% confidence interval (CI), 1.1–5.4), whereas there was no association in older girls.

## 4. Discussion

There are several noticeable findings in the current study, which analyzed a nationwide sample of Korean adolescent boys and girls. First, we documented perceptible evidence on the independent association between relative HG strength and high cardiometabolic risk in adolescents, while considering age, sex, body weight, height, current smoking, alcohol drinking, and sedentary time. Second, CMRI score, which discriminates between favorable and unfavorable metabolic profiles and relative HG strength, can be a valuable indicator of the risk of cardiometabolic disease in adolescents. Finally, smoking, an established risk factor for cardiometabolic disease in adults, was also associated with high cardiometabolic risk in boys aged 15 to 18 years.

Our study demonstrated an independent negative association between HG strength and cardiometabolic risk in a large population of adolescents. Previous studies involving adolescents on the association between HG strength and cardiometabolic risk lacked confounder control [5,20,33]. Furthermore, the results of our study suggest that the lower the relative HG strength, the higher the cardiometabolic risk. The adolescents in the lowest quartile of relative HG strength showed higher ORs for high cardiometabolic risk than those in the second quartile, with the highest quartile (most strong adolescents) as the reference group. The adolescents in the third quartile of relative HG strength showed similar cardiometabolic risk as those in the highest quartile. Considering these dose-response relationships, a cut-point of relative HG strength that can predict high cardiometabolic risk in adolescents can be obtained. Future studies using ROC analysis can determine the cut-points of HG strength to predict high cardiometabolic risk for the Korean population.

The results of this study are in agreement with the results of the FUPRECOL study of Colombia, i.e., relative HG strength may be helpful as an effective screening tool for adolescents with high cardiometabolic risk [5]. However, there are many confounding factors to be considered in the pediatric population. Because adolescence is the period in which growth and development are still in progress, the effect of numerical age difference is not the same as that in adults. In addition, rapid growth accompanied by secondary sexual development can have a substantial effect on the association between muscle strength and cardiometabolic risk [34,35]. Therefore, developing a growth chart for relative HG strength can be better than using unified cutoff values by receiver operating curves to predict the cardiometabolic risk across adolescents in different age groups.

We found that both boys and girls with high cardiometabolic risk, as defined by the CMRI score, have poor metabolic features, such as obesity, high blood pressure and fasting glucose level, and low HDL-C level. These findings are consistent with the report by Cohen et al., which showed that poor relative HG strength was associated with a poor metabolic risk profile [19]. Interestingly, the mean HG strength was higher, but the mean relative HG strength was lower in participants with high cardiometabolic risk than in those with low risk. Relative HG strength is a body weight-normalized value reflecting the weight growth of an adolescent, possibly related to the quality of the muscles. Therefore, relative HG strength may be a better indicator of cardiometabolic risk than HG strength. The results of our study reinforce the idea that weak muscle strength and/or poor muscle quality of adolescents could lead to unfavorable cardiometabolic health outcomes in adolescence and adulthood [2,13].

The prevalence of metabolic syndrome in adolescence ranges from 0.2% to 38.9%, depending on the definition applied and the population examined [3]. The prevalence of metabolic syndrome in our study (2.7%) was comparable to that in a previous study in Korean adolescents [20]. There are no globally accepted diagnostic criteria for metabolic syndrome in the pediatric population because each component of the syndrome has different reference ranges according to age, sex, and ethnicity. Moreover, insulin resistance is considered normal in adolescence [36] but is considered abnormal in adulthood. The CMRI is the sum of clustered cardiometabolic risk components, which has been validated for use in children and adolescents [5,6,7,8]. The high sensitivity and specificity of high cardiometabolic risk defined by the CMRI score to detect metabolic syndrome in the current study support the fact that the CMRI score is an accurate index to screen cardiometabolically unhealthy adolescents.

Smoking behavior has been regarded as an important risk factor for cardiometabolic diseases in adolescents as well as in adults [23]. Nevertheless, there was no significant association between smoking behavior and cardiometabolic risk in the present study analyzing the whole age range of adolescence. After age was dichotomized, in older boys, the currently smoking boys had a 2.5-fold higher OR of high cardiometabolic risk than non-smoking boys. Our study suggests that adolescent smoking has an independent association with the cardiometabolic risk even after adjusting for muscular strength and other confounders in older boys. This result suggests the need for targeted public health programs to reduce smoking among boys aged 15–18 years.

The HG and relative HG strength values in adolescents according to age and sex reported in the current study should be acknowledged. Our finding that boys are stronger than girls in all age-groups is consistent with the findings of previous studies that suggested innate sex differences in muscle strength [32]. Relative HG strength increased with age from the age of 12 years until 14 in boys but remained similar in girls of all ages. This gender difference in relative HG strength change according to age may be explained by the different onset of puberty in boys and girls, which can affect body composition and muscular growth patterns [34,35]. After puberty, when sex hormones begin secreting testosterone, there can be different development of the skeletal muscle mass between the sexes. Because puberty begins and reaches completion earlier in girls than in boys, girls may reach the maximum level of relative HG strength earlier than boys.

The strength of the current study is the adjustment of the multiple confounders to evaluate the independent association between HG strength, an objective measure of muscle strength, and cardiometabolic risk in adolescents. Additionally, HG strength was evaluated in a large number of adolescents, representing the entire Korean adolescent population. Because we used complex-sample statistics reflecting the sampling weights, our findings can be interpreted as the results of the entire Korean adolescents. However, there are some limitations to the current study. First, because of the cross-sectional design of the survey, establishing a causal relationship between muscle strength and cardiometabolic risk was difficult. Second, we could not identify the exact number of participants who were unable to give HG strength measurements and the reason behind this. Third, although clinically important potential covariates were included in the regression models, some of the results may be confounded by unmeasured variables. Fourth, the ORs of the current study should be interpreted with caution because ORs can overstate the effect size when interpreted as a relative risk. Lastly, we could not moderate the pubertal development stage to muscle strength and physical activity level because these data were unavailable in KNHANES during the study period.

## 5. Conclusions

Relative HG strength showed an independent negative association with high cardiometabolic risk in adolescents. The stronger the relative HG strength, the lower the cardiometabolic risk. The CMRI can be a valuable indicator of the risk of cardiometabolic diseases in adolescents with poor anthropometric and biochemical profiles. Public management to reduce smoking in older adolescent boys may be an important approach to reduce the cardiometabolic risk in this population. Future studies should define age- and sex- specific cut-points of relative HG strength with adjustment for confounders to predict high cardiometabolic risk.

## Figures and Tables

**Table 1:** Clinical characteristics, biochemical profile, handgrip strength, and sedentary time of adolescents according to cardiometabolic risk.

Cardiometabolic Risk	Boys (*n* = 952)			Girls (*n* = 854)		
	Low (*n* = 809)	High (*n* = 143)	*p* *	Low (*n* = 740)	High (*n* = 114)	*p* *
Age (years)	15.3 ± 0.1	15.2 ± 0.2	0.477	15.2 ± 0.1	15.1 ± 0.2	0.813
Height (cm)	169.8 ± 0.3	170.8 ± 0.6	0.159	159.7 ± 0.2	161.2 ± 0.6	0.017
Body weight (kg)	60.7 ± 0.5	77.3 ± 1.4	<0.001	52.6 ± 0.4	66.0 ± 1.4	<0.001
Body mass index (kg/m^2^)	21.0 ± 0.1	26.3 ± 0.4	<0.001	20.6 ± 0.1	25.3 ± 0.5	<0.001
Weight status			<0.001			<0.001
Underweight	10.9 (1.3)	1.5 (0.9)		7.2 (1.2)	0.0 (0.0)	
Healthy weight	75.0 (1.6)	30.4 (4.5)		77.0 (1.8)	37.1 (4.9)	
Overweight	7.4 (1.0)	19.3 (3.3)		8.1 (1.1)	17.3 (4.0)	
Obesity	6.6 (1.0)	48.8 (4.6)		7.7 (1.1)	45.6 (5.3)	
Waist circumference (cm)	72.4 ± 0.3	87.7 ± 1.0	<0.001	68.1 ± 0.3	79.1 ± 1.1	<0.001
SBP (mmHg)	109.9 ± 0.4	121.9 ± 0.8	<0.001	104.9 ± 0.4	115.6 ± 1.2	<0.001
DBP (mmHg)	66.7 ± 0.3	74.3 ± 0.7	<0.001	65.7 ± 0.3	73.1 ± 0.8	<0.001
Fasting glucose (mg/dL)	91.9 ± 0.3	96.4 ± 0.7	<0.001	88.9 ± 0.3	96.5 ± 1.3	<0.001
Total cholesterol (mg/dL)	152.9 ± 1.0	168.3 ± 2.8	<0.001	166.4 ± 1.1	175.7 ± 2.5	0.001
HDL-C (mg/dL)	50.9 ± 0.3	41.7 ± 0.7	<0.001	54.6 ± 0.4	44.8 ± 0.8	<0.001
TG (mg/dL)	75.1 ± 1.4	151.0 ± 8.5	<0.001	76.9 ± 1.3	138.4 ± 6.3	<0.001
CMRI score	−1.012 ± 0.094	5.684 ± 0.181	<0.001	−0.912 ± 0.098	5.983 ± 0.316	<0.001
Current smoking (%)	9.1 (1.2)	13.3 (3.7)	0.223	2.9 (0.7)	3.1 (1.8)	0.891
Alcohol drinker (%)	9.5 (1.3)	10.9 (3.3)	0.674	6.4 (1.0)	6.3 (2.4)	0.971
Handgrip strength (kg)	31.4 ± 0.3	33.5 ± 0.7	0.008	21.2 ± 0.2	22.7 ± 0.5	0.006
Relative handgrip strength	0.522 ± 0.004	0.442 ± 0.012	<0.001	0.407 ± 0.004	0.353 ± 0.007	<0.001
Sedentary time (min/day)	634.1 ± 6.7	655.5 ± 15.9	0.217	681.5 ± 7.8	642.4 ± 17.8	0.052

Values are weighted means ± standard errors or weighted percentage (standard errors), as appropriate. High cardiometabolic risk was defined as ≥1 standard deviation of the cardiometabolic risk index score. * *p* values comparing high and low cardiometabolic risk by Student’s *t*-tests or the Chi square tests, as appropriate. SBP, systolic blood pressure; DBP, diastolic blood pressure; HDL-C, high-density lipoprotein cholesterol; TG, triglycerides.

**Table 2:** Odds ratios for the association of relative handgrip strength and smoking behavior with high cardiometabolic risk.

Variable	Boys (*n* = 952)		Girls (*n* = 854)	
	Unadjusted	Adjusted *	Unadjusted	Adjusted *
Relative handgrip strength ^†^				
Most weak quartile (Q1)	8.1 (3.5–18.8) ^‡^	8.5 (3.7–19.3) ^‡^	5.7 (3.0–11.1) ^‡^	5.7 (2.9–11.2) ^‡^
Second quartile (Q2)	3.9 (1.7–9.2) ^‡^	3.9 (1.7–8.9) ^‡^	2.6 (1.3–5.2) ^‡^	2.6 (1.3–5.3) ^‡^
Third quartile (Q3)	1.8 (0.7–4.5)	1.8 (0.8–4.5)	1.1 (0.5–2.6)	1.1 (0.5–2.6)
Highest quartile (Q4)	Reference	Reference	Reference	Reference
Current smoking	1.5 (0.8–3.0)	1.9 (0.9–4.0)	1.1 (0.3–3.8)	1.7 (0.5–6.4)

Values are the odds ratios (95% confidence intervals); High cardiometabolic risk was defined as ≥1 standard deviation of cardiometabolic risk index score; * Adjusted for height, sedentary time, alcohol consumption, and all variables in the same column; ^†^ Age- and sex-specific quartiles; ^‡^ *p* < 0.05 by complex-sample logistic regression models.

**Table 3:** Odds ratios for the association of relative handgrip strength and smoking behavior with high cardiometabolic risk according to age groups.

Variable	Boys (*n* = 952)		Girls (*n* = 854)	
	12–14 Years * (*n* = 427)	15–18 Years * (*n* = 525)	12–14 Years * (*n* = 380)	15–18 Years * (*n* = 474)
Relative handgrip strength ^†^				
Most weak quartile (Q1)	7.9 (2.8–22.6) ^‡^	10.1 (3.5–29.2) ^‡^	4.5 (1.9–11.0) ^‡^	7.3 (2.6–20.4) ^‡^
Second quartile (Q2)	7.2 (2.6–19.6) ^‡^	3.0 (0.99–9.3)	1.8 (0.7–4.8)	3.8 (1.3–11.0) ^‡^
Third quartile (Q3)	3.7 (1.4–10.2) ^‡^	1.2 (0.3–4.3)	0.8 (0.2–2.6)	1.5 (0.4–5.3)
Highest quartile(Q4)	Reference	Reference	Reference	Reference
Current smoking	-	2.5 (1.1–5.4) ^‡^	-	2.0 (0.5–7.6)

Values are the odds ratios (95% confidence intervals); High cardiometabolic risk was defined as ≥1 standard deviation of the cardiometabolic risk index score; * Adjusted for height, sedentary time, alcohol consumption, and all other variables in the same column; ^†^ Age- and sex-specific quartiles; ^‡^ *p* < 0.05 by complex-sample logistic regression models.

## Supplementary Materials

The following are available online at https://www.mdpi.com/1660-4601/17/14/5021/s1, Figure S1. Flow diagram of adolescents included and excluded in the present study. KNHANES, Korea National Health and Nutrition Examination Surveys; CMRI, cardiometabolic risk index. Table S1. Mean handgrip and relative handgrip strength according to age and sex.
