# Supplementary material for: Handgrip Strength and Current Smoking Are Associated with Cardiometabolic Risk in Korean Adolescents: A Population-Based Study
Source: Int J Environ Res Public Health. 2020 Jul 13;17(14):5021. doi: 10.3390/ijerph17145021 (PMC7400210; doi:10.3390/ijerph17145021)
Supplement: Supplementary file 1 [file ijerph-17-05021-s001.pdf]

Figure S1.

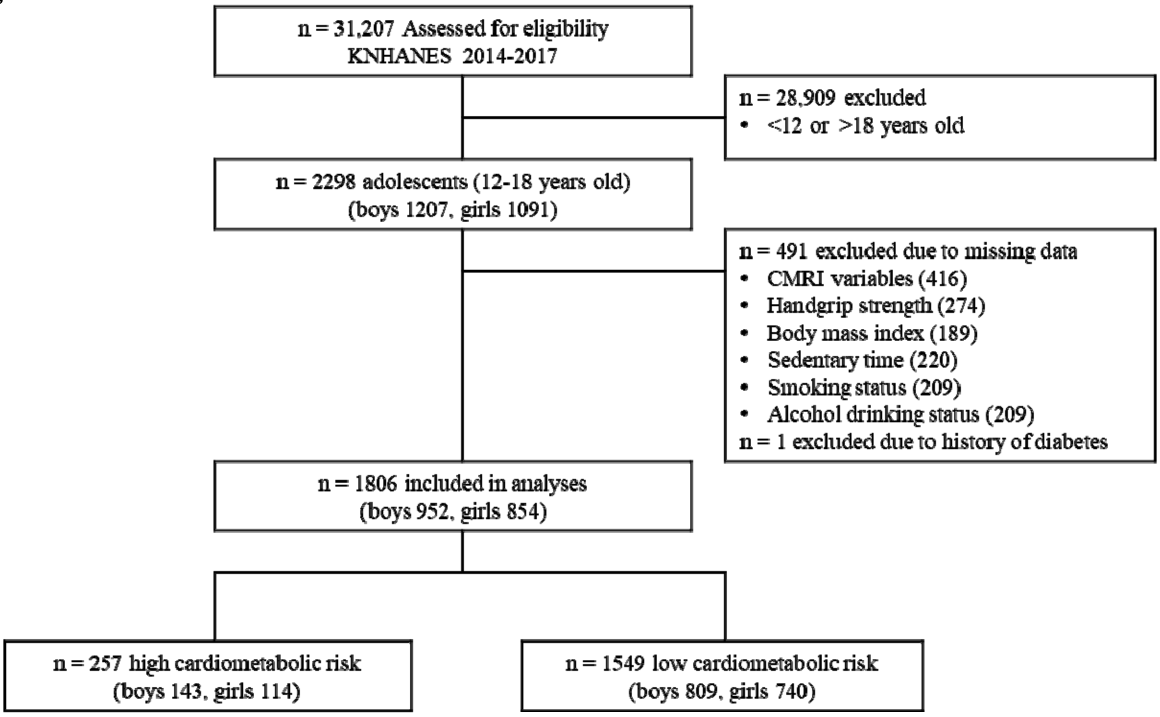

Table S1. Mean handgrip and relative handgrip strength according to age and sex.

	Number		Handgrip strength (kg)		Relative handgrip strength	
Age (years)	Boys	Girls	Boys	Girls	Boys	Girls
12	126	120	20.8±0.5[†]	18.5±0.4[†]	0.426±0.009[†]	0.389±0.007
13	165	133	26.8±0.5[†]	20.5±0.4[†]	0.471±0.008[†]	0.408±0.007
14	136	127	31.1±0.7[†]	20.1±0.3[†]	0.505±0.011[†]	0.391±0.007
15	142	117	32.8±0.5[†]	21.8±0.4	0.512±0.010	0.403±0.008
16	129	131	34.6±0.7	22.5±0.4	0.537±0.011	0.407±0.009
17	125	129	35.4±0.6	22.8±0.4	0.542±0.010	0.402±0.009
18	129	97	35.4±0.6	22.3±0.5	0.536±0.010	0.396±0.010
Total	952	854	31.7±0.3	21.4±0.2	0.510±0.004	0.400±0.003
P for trend[*]			<0.001	<0.001	<0.001	0.306

Values are the weighted means ± standard errors.
[*]*P* for trend values by complex-sample general linear models.
[†]*P* < 0.05 by *post hoc* analyses, age 18 years old as reference.
